# Novel Potential Biomarkers for Retinopathy of Prematurity

**DOI:** 10.3389/fmed.2022.840030

**Published:** 2022-02-02

**Authors:** Wei Tan, Bingyan Li, Zicong Wang, Jingling Zou, Yang Jia, Shigeo Yoshida, Yedi Zhou

**Affiliations:** ^1^Department of Ophthalmology, The Second Xiangya Hospital of Central South University, Changsha, China; ^2^Hunan Clinical Research Center of Ophthalmic Disease, Changsha, China; ^3^Department of Pediatrics, The Second Xiangya Hospital of Central South University, Changsha, China; ^4^Department of Ophthalmology, Kurume University School of Medicine, Kurume, Japan

**Keywords:** biomarker, retinopathy of prematurity, metabolites, cytokines and growth factors, non-coding RNAs, gut microbiota, oxidative stress biomarkers, iconography

## Abstract

Retinopathy of prematurity (ROP) is the main risk factor for vision-threatening disease in premature infants with low birth weight. An accumulating number of independent studies have focused on ROP pathogenesis and have demonstrated that laser photocoagulation therapy and/or anti-VEGF treatment are effective. However, early diagnosis of ROP is still critical. At present, the main method of ROP screening is based on binocular indirect ophthalmoscopy. However, the judgment of whether ROP occurs and whether treatment is necessary depends largely on ophthalmologists with a great deal of experience. Therefore, it is essential to develop a simple, accurate and effective diagnostic method. This review describes recent findings on novel biomarkers for the prediction, diagnosis and prognosis of ROP patients. The novel biomarkers were separated into the following categories: metabolites, cytokines and growth factors, non-coding RNAs, iconography, gut microbiota, oxidative stress biomarkers, and others. Biomarkers with high sensitivity and specificity are urgently needed for the clinical applications of ROP. In addition, using non-invasive or minimally invasive methods to obtain samples is also important. Our review provides an overview of potential biomarkers of ROP.

## Introduction

Retinopathy of prematurity (ROP) is a major cause of vision loss and blindness in children worldwide (1–3). As perinatal oxygen metabolism disorder causes hypoxia-ischemia, the compensatory secretion of pathological angiogenic factors and then the formation of retinal neovascularization contributes to abnormal retinal blood vessel development and even tractional retinal detachment (4–6). Preterm infants have immature retinal tissue, shorter axial lengths and thicker corneas and are more likely to suffer from ROP (7, 8). Epidemiological studies have shown that the incidence of premature newborns is approximately 10%, and infants with lower birth weight and gestational age have a higher incidence and severity of ROP. A total of 65.8% of preterm infants with a birth weight of <1,251 g suffer from ROP to a certain degree; 81.6% of infants with a birth weight of <1,000 g suffer from ROP (9, 10). However, ROP is a largely preventable disease. Reducing the incidence of blindness is related to high-quality newborn care, a comprehensive ROP screening program, and experienced ROP ophthalmologists (11). Laser photocoagulation combined with intravitreal injection of anti-vascular endothelial growth factor (VEGF) drugs after early detection is effective (12–14).

At present, ROP screening is based on birth weight and gestational age. Binocular indirect ophthalmoscopy (BIO) bedside examination, as well as wide-field fundus imaging system, are widely applied for ROP screening (15, 16). Recently, telemedicine and artificial intelligence-based ROP screening are considered to be more suitable in remote areas that are lack trained ophthalmologists (16–18). However, these screening methods have not been widely implemented, and the judgment of whether the therapeutic treatment is required for ROP infants mainly depends on the clinical experiences of the ophthalmologists (19–21). Furthermore, the molecular diagnostic methods and diagnostic criteria of ROP have not yet been clarified. A balance between the accurate identification of newborns with ROP that need therapy and a reduction in workload is required to save resources and avoid the unnecessary inspection of prematurity (22).

**Figure 1:**
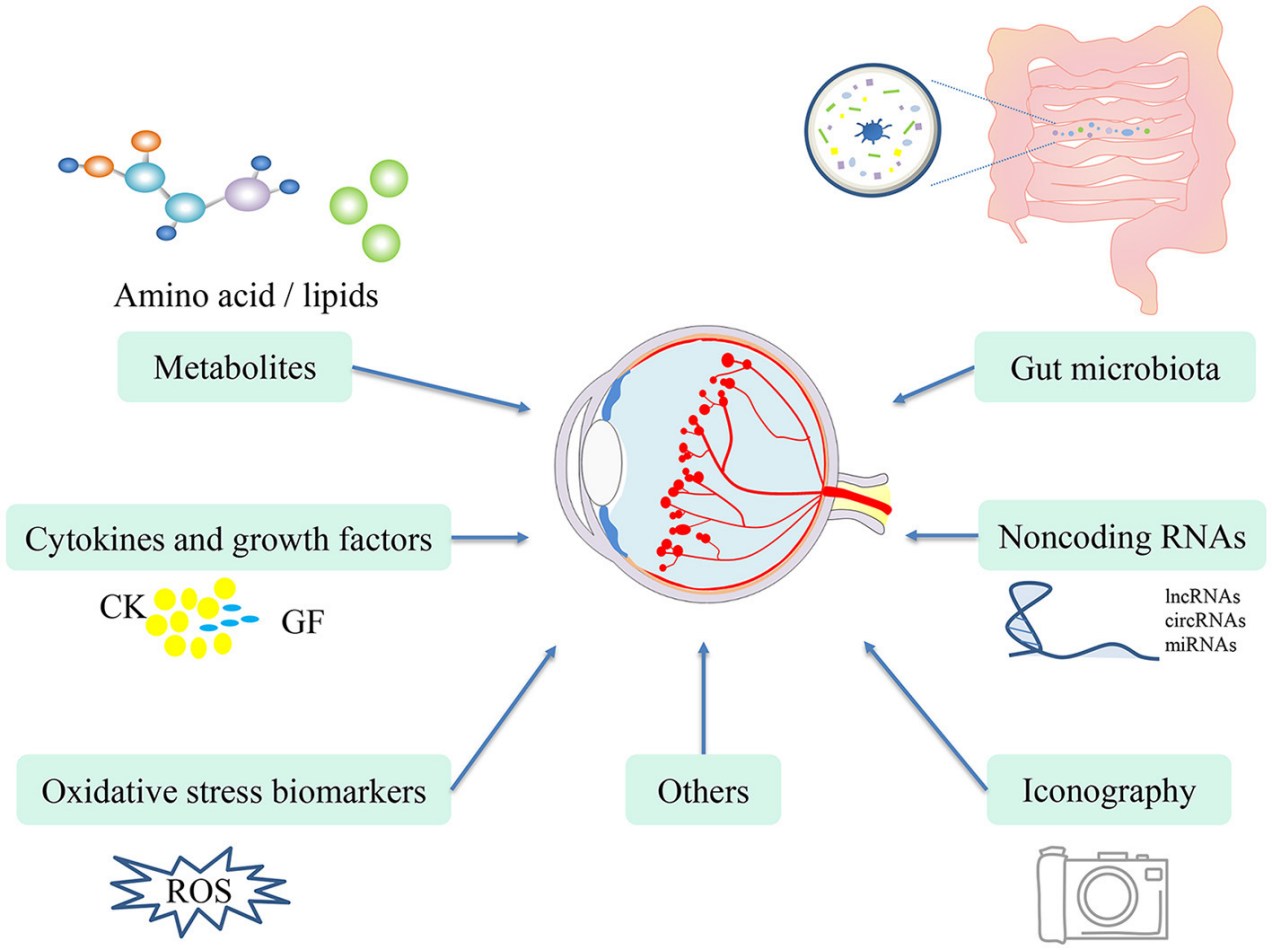
Novel potential biomarkers for ROP, including metabolites, cytokines and growth factors, ncRNAs, iconography, gut microbiota, oxidative stress biomarkers and others.

Biomarkers are indicators that are defined as objective measurements and evaluations that are used to evaluate normal biological processes, pathogenic processes, and responses to intervention or exposure (23). Biomarkers are mainly divided into seven categories, including diagnosis, monitoring, drug efficacy/response, prediction, prognosis, safety and susceptibility/risk biomarkers (23, 24). Blood is easy to obtain, and blood draw is a relatively non-invasive method (25). Some diseases have been distinguished using blood-based biomarkers, such as human epidermal growth factor receptor 2 (HER2) for breast cancer (26, 27) and epidermal growth factor receptor (EGFR) for lung cancer (28, 29). In addition to blood biomarkers, biomarkers from urine, feces and cerebrospinal fluid can be used to identify diseases. One such biomarker in the spinal fluid is myelin oligodendrocyte glycoprotein (MOG-IgG) and aquaporin-4 (AQP4), which are used to identify neuromyelitis optica spectrum disorders (30). In the field of ophthalmology, imaging findings and artificial intelligence analysis have been used as biomarkers for prediction and therapy response of choroidal diseases (31–33), and intraretinal cysts can be used for the prognosis of neovascular age-related macular (AMD) disease (34). Our review provides an overview of biomarkers in ROP and summarized in Figure 1.

## Candidates of Novel Potential Biomarkers

### Metabolites

ROP is related to hypoxia and nutrient deprivation in the maturation of retinal blood vessels, and regulation of retinal metabolism can prevent pathological angiogenesis. Recent research on the metabolic changes of ROP shows that metabolites can serve as biomarkers (35). Metabolomics affects cell physiology by regulating the genome, epigenome, transcriptome, and proteome (36). A study on targeted blood metabolomics in premature neonates showed that elevated levels of malonyl carnitine (C3DC) and glycine in the blood are promising biomarkers for prediction but cannot judge severity (37). Another plasma metabolomics study on treatment-requiring ROP indicated that altered metabolites may be used as diagnostic and prognostic biomarkers, including the majority of altered amino acids and their derivatives (38). Further targeted metabolomics research found that plasma citrulline, arginine and aminoadipate were increased in patients with ROP, but creatine was reduced. They are all potential biomarkers (39). The oxygen-induced retinopathy (OIR) model is an animal model that has been widely used in the study of retinal neovascular diseases and is similar to the pathological process of ROP (40, 41). By analyzing the plasma from an OIR rat model, Lu et al. found that proline and “arginine and proline metabolism” pathways are potential biomarkers for the diagnosis of ROP (42). A prospective study by Nilsson et al. analyzed the changes in serum sphingolipidome in very preterm infants and concluded that a low concentration of sphingosine-1-phosphate signaling lipid is strongly related to severe ROP (43). Lower levels of the ω-6 long-chain polyunsaturated fatty acid arachidonic acid (AA) are closely related to the development of ROP and is beneficial for the prediction of ROP (44). Table 1 summarizes these metabolism biomarkers.

**Table 1:** Metabolites as potential biomarkers for ROP patients.

**Sample source**	**Method**	**Potential biomarker**	**Correlation**
Infant-blood	Targeted metabolomic	C3DC and glycine	Elevated levels of C3DC and glycine in premature infants are promising biomarkers for ROP prediction, not for severity (37).
Infant-plasma	Untargeted metabolomics analysis	altered metabolites	Altered metabolites may be used as diagnostic and prognostic biomarkers, especially altered amino acids and their derivatives (38).
Infant-plasma	Targeted metabolomics analysis	citrulline, arginine,aminoadipate and creatine	Citrulline, arginine and aminoadipate in patients with ROP were increased, while creatine was reduced (39).
OIR rat-plasma	Untargeted metabolomics analysis	proline and “arginine and proline metabolism” pathways	Proline and “arginine and proline metabolism” pathways are potential biomarkers for the diagnosis of ROP (42).
Infant-serum	Lipid analysis	sphingosine-1-phosphate	Low levels of the sphingosine-1-phosphate signaling lipid is strongly related to severe ROP (43).
Infant-serum	Lipid analysis	AA	Low levels of AA are closely related to the development of ROP and benefit prediction (44).

### Cytokines and Growth Factors

The immune-inflammatory environment before and after delivery of preterm infants may be a crucial factor leading to the progression of ROP (45). Various inflammatory cytokines and chemokines have been extensively investigated, and changes in their levels may be potential candidates for novel biomarkers of ROP. A study indicated that the increase in inflammatory factors (interleukin (IL)-6 and IL-8) and angiogenic mediators (endoglin, endostatin and insulin-like growth factor-binding protein (IGFBP)-2) in amniotic fluid is related to the occurrence and development of ROP. The use of these biomarkers in combination with prenatal factors can establish a prenatal prediction pattern of ROP (46). Another similar study suggested that an increase in IL-6 in umbilical cord plasma can predict ROP severity, and the elevated concentration of C5a can be used to assess whether laser treatment is required. Furthermore, the combined application is more accurate in the prediction of ROP development (45). In umbilical cord serum, elevated levels of IL-7, monocyte chemotactic protein-1 (MCP-1), macrophage inflammatory protein 1α (MIP-1α) and MIP-1β contribute to predicting the risk of ROP, while MIP-1β is related to ROP severity (47). High levels of VEGF-Receptor 1, IL-8, matrix metalloproteinase 9 (MMP-9), erythropoietin (EPO), tumor necrosis factor (TNF)-α and basic fibroblast growth factor (bFGF) are related to a risk factor for prethreshold ROP in the first three postnatal weeks (48). A similar study showed that IL-6 is significantly increased and IL-17 is decreased on Days 0–3 after birth. On Days 7–21, transforming growth factor-β (TGF-β), brain-derived neurotrophic factor (BDNF), and regulated upon activation, normal T cell expressed and secreted (RANTES) were significantly reduced. IL-18, CRP and NT-4 concentrations were changed in both time periods (49). On Day 28, elevated concentrations of IL-6, TNF-α, TNF-R1/-R2, and IL-8 were related to the risk of ROP (48), and decreased serum levels of EPO was determined to be an independent factor for ROP prediction (50). In the tears of severe ROP, MMP-9 is elevated. Moreover, in the ROP vitreous, MMP9, complement factor H (CFH), C3, C4, IL-1ra, VEGF and granulocyte colony-stimulating factor (G-CSF) are also increased (51). VEGF is elevated, and insulin-like growth factor 1 (IGF-1) is reduced in the cord blood of ROP patients. Serum IL-33 and endocan could be predictive biomarkers for severe ROP. These serum concentrations were higher and then significantly reduced after laser treatment (52). VEGF, interferon-γ (IFN-γ), IL-10 and IL-12 are elevated in the aqueous humor of ROP patients, and higher levels of VEGF and MIP-1β are independently associated with ROP retreatment (53). At birth, infants with proliferative ROP have a low level of serum IL-5. Ten to 14 days after birth, babies without ROP have higher levels of serum BDNF and RANTES than infants with proliferative ROP (54). At 24 h after birth, the levels of proinflammatory cytokines (IL-6 and TNF-α) were increased in children who received ROP therapeutic treatment. However, the concentration of IL-6 was negatively correlated with IGF-1 in ROP infants of 5–8 weeks after birth (55).

IGF-1 is the primary factor involved in the growth of fetal tissues. Under normal circumstances, sufficient levels of serum IGF-1 are required for VEGF-stimulated retinal angiogenesis, but premature delivery causes a sudden decrease in serum IGF-1 levels (56, 57). Clinical studies in the United States (56) and Europe (58) have shown that a low concentration of IGF-1 is related to the subsequent progression of severe ROP, so it is a risk predictor. The early return of IGF-1 to normal levels in premature infants can prevent ROP (59). The critical time for the detection of serum IGF-1 is in the third week after delivery (57). Visfatin is an adipocytokine that has a similar to insulin function and IGF-1 level, and it could be considered to be a predictor of ROP (60). Serum VEGF levels at birth are reduced in premature newborns who develop ROP later and may be an ROP predictor (61). VEGF and stromal cell-derived factor 1α (SDF-1α) are elevated in the vitreous of stage 4 ROP (62). Among the proangiogenic factors in infant tears, angiogenin/VEGF can be used as a potential non-invasive screening biomarker for ROP (63). However, Woo et al. found that inflammatory cytokines (IL-1β, IL-4, IL-6, IL-8, IL-10, IL12, IFN-γ, and TNF-α) and growth factors (IGF-1 and VEGF) in cord blood samples may not predict ROP (64). Lymphocyte count is negatively correlated with ROP and may have an independent predictive value. However, the neutrophil-to-lymphocyte ratio (NLR) is not an independent predictor of ROP (65). Table 2 summarizes the cytokine and growth factor biomarkers.

**Table 2:** Cytokines and growth factors as potential biomarkers for ROP patients.

**Sample source**	**Method**	**Potential biomarker**	**Correlation**
Infant-amniotic fluid	ELISA	IL-6, IL-8, endoglin, endostatin and IGFBP-2	Inflammatory factors (IL-6 and IL-8) and angiogenic mediators (endoglin, endostatin and IGFBP-2) in amniotic fluid are related to the occurrence and development of ROP (46).
Infant-plasma	ELISA	IL-6 and C5a	High IL-6 levels predict ROP severity, while elevated concentrations of C5a assess whether laser treatment is required. The combined application is more accurate in predicting ROP development (45).
Infant-serum	multiplex protein arrays	IL-7, MCP-1, MIP-1α and MIP-1β	Elevated levels of IL-7, MCP-1, MIP-1α and MIP-1β contribute to prediction of the risk of ROP, and MIP-1β is related to ROP severity (47).
Infant-blood	meso scale discovery multiplex platform and microplate detection platform	VEGF-R1, IL-8, MMP-9, EPO, TNF-α and bFGF	High levels of VEGF-R1, IL-8, MMP-9, EPO, TNF-α and bFGF are related to a risk factor for prethreshold ROP in the first three postnatal weeks. On Day 28, elevated concentrations of IL-6, TNF-α, TNF-R1/-R2, IL-8 are still related to the risk (48).
Infant-blood	Multiplex Luminex assay	IL-6, IL-17, TGF-β, BDNF, RANTES, IL-18, CRP and NT-4	IL-6 is significantly increased and IL-17 is decreased on Days 0–3 after birth. On Days 7–21, TGF-β, BDNF, and RANTES are significantly reduced. IL-18, CRP and NT-4 were changed in both time periods (49).
Infant-serum	ELISA	EPO	On Day 28, decreased serum levels of EPO were determined to be independent factors for ROP prediction (50).
Infant-vitreous and tear	multiplex bead arrays	MMP9, CFH, C3, C4, IL-1ra, VEGF and G-CSF	In tears of severe ROP, MMP-9 is elevated. In the ROP vitreous, MMP9, CFH, C3, C4, IL-1ra, VEGF and G-CSF are also increased (51).
Infant-blood	ELISA	VEGF, IGF-1, IL-33 and endocan	VEGF is elevated and IGF-1 is reduced in cord blood of ROP patients. Serum IL-33 and endocan could be predictive biomarkers for severe ROP (52).
Infant-aqueous humor	multiplex bead assay	VEGF, IFN-γ, IL-10, IL-12 and MIP-1β	VEGF, IFN-γ, IL-10 and IL-12 in ROP patients are elevated, and higher levels of VEGF and MIP-1β are independently associated with ROP retreatment (53).
Infant-serum	multiplex immunoassay	IL-5, BDNF and RANTES	Infants at birth with proliferative ROP have a low level of serum IL-5. Ten to 14 days after birth, babies without ROP have higher levels of serum BDNF and RANTES than infants with proliferative ROP (54).
Infant-blood	human Luminex xMAP assay	IL-6, TNF-α and IGF-1	At 24 h after birth, the levels of IL-6 and TNF-α are both increased in children who received ROP treatment, while the concentration of IL-6 is negatively correlated with IGF-1 between 5–8 weeks after birth (55).
Infant-serum	ELISA and IGF binding protein-blocked radioimmunoassay	IGF-1	A low concentration of IGF-1 is related to the subsequent progression of severe ROP, so it is a risk predictor (56, 58). In addition, the early return of IGF-1 to normal levels in premature infants can prevent ROP (59). The critical time for the detection of serum IGF-1 is in the third week after delivery (57).
Infant-blood	Enzyme immunoassay	IGF-1 and Visfatin	Visfatin is an adipocytokine that has a similar to insulin function and IGF-1 level, and could be considered a predictor of ROP (60).
Infant-blood	ELISA	VEGF	Serum VEGF levels are reduced in premature newborns who develop ROP, and may be a predictor of ROP (61).
Infant-vitreous	ELISA	VEGF and SDF-1α	VEGF and SDF-1α are elevated in the vitreous of stage 4 ROP (62).
Infant-tear fluid	multiplex ELISA	Angiogenin and VEGF	Among the pro-angiogenic factors in the tears of infants, angiogenin/VEGF may be a potential non-invasive screening biomarker for ROP (63).
Infant-blood	ABX Pentra DF120/USA biochemical analyzer	Lymphocyte count	Lymphocyte count is negatively correlated with ROP and may be an independent predictor (65).

### Non-coding RNA

Non-coding RNAs (ncRNAs) are a type of RNA that does not encode proteins (66). However, they can still affect normal gene expression and participate in physiological and pathological processes through various mechanisms (67–69). Types of ncRNAs include long non-coding RNAs (lncRNAs), circular RNAs (circRNAs), and microRNAs (miRNAs), etc. (70). CircRNA is a single-stranded closed circular ncRNA. Compared to other RNAs, it has a longer half-life, better stability and increased resistance to RNase R, making it a potential biomarker and therapeutic target (71–73). In fact, the expression and function of various circRNAs have been indicated in different cancers (74) and ocular diseases (75). Based on an OIR mouse model, Liu et al. found that the expression of cZNF609 in the retina was largely reduced during the vascular occlusion phase and significantly increased during the neovascularization phase. It combines with miR-615-5p as a miRNA sponge to regulate the gene expression of human umbilical vein endothelial cells (76). MiRNAs are small ncRNAs that can influence gene expression by influencing transcription, translation and epigenetics (77). At present, most studies on miRNAs and ROP are derived from research in animal models (78, 79). Metin et al. (80) performed the first clinical study. Through the analysis of 13 cases of ROP and 15 cases of premature infants without ROP, they found that miR-23a and miR-200b-3p were significantly elevated in premature infants with ROP, while miR-27b-3p and miR-214-3p were reduced. These altered miRNAs could be considered as possible biomarkers of ROP (80). Furthermore, there are some related transcriptomics and bioinformatics analyses that can also provide novel for ncRNA as ROP biomarkers (81–83).

### Oxidative Stress Biomarkers

ROP is a neonatal disease that is associated with oxidative stress. When a premature baby is born, it suddenly changes from a very low oxygen intrauterine environment to an artificial hyperoxia treatment environment. Due to an insufficient antioxidant protection capacity, it is in a state of oxidative stress, and the retina is particularly sensitive to oxidative stress (84, 85). The glutathione status of red blood cells is an indicator of oxidative stress in preterm infants, and it aids in the early identification of children at risk of ROP (86). The acrolein-lysine adduct was elevated in the premature infant group with active retinopathy compared with the preterm group without retinopathy (87).

The levels of 8-hydroxy 2-deoxyguanosine (8-OHdG) and malondialdehyde (MDA) are significantly higher in the blood and urine of ROP patients than in non-ROP patients. Based on this correlation, they could be used as screening indicators for ROP (88). In umbilical cord plasma, elevated levels of the oxidative stress biomarker MDA and reduced levels of the micronutrient vitamin A in infants are independent predictors of ROP (89). Other studies also recognized that total oxidative status (TOS) and MDA are satisfactory markers of oxidative stress, which is increased in the ROP group (90). Peroxidant antioxidant balance (PAB) contributes to the incidence of ROP, and the severity of ROP increases with PAB (91).

### Gut Microbiome

Although the human intestine is far from the eye, ophthalmological diseases are related to the regulation of systemic immunity. Emerging investigations into changes in the gut microbiota have been reported with a focus on uveitis (92, 93) and AMD (94, 95), and the concept of the gut-retina axis has emerged (94, 96). The maternal gut microbiota plays a key role in the health of infants (97). We can screen out differentially expressed gut microbes and explore the potential biomarkers of ROP. Such biomarkers could have important clinical significance and application value for the preliminary screening of certain concealed and difficult-to-diagnose ROPs. Westaway et al. suggested that preterm birth-related diseases are associated with the gut microbiome and that α-diversity in ROP infants was significantly reduced (98). Other studies proposed that Enterobacteriaceae species are enriched a few weeks before the diagnosis of ROP, while amino acid synthesis is more abundant in the non-ROP group (99, 100). Changes in the intestinal flora are promising targets for prevention and therapy in ROP patients. Such changes are closely related to metabolites. Therefore, it might be effective to utilize beneficial bacteria or produce antibodies against pathogenic bacteria to prevent or treat ROP in infants.

### Iconography

In some remote regions, telemedicine technology with a digital fundus camera has been used for the screening and diagnosis of ROP (101). The swept-source optical coherence tomography imaging is used to determine the choroidal vascularity index (CVI), which is more sensitive than subfoveal choroidal thickness in assessing related choroidal structural changes in premature infants with a history of ROP. A decrease in CVI indicates impaired choroidal vascular function (102).

### Other Biomarkers

There is significant thrombocytopenia in the blood samples of infants with treatment-requiring ROP, and this could be a predictor of disease progression (103). Platelet mass index is a reliable monitoring indicator for the prognosis of ROP in very premature newborns (104). The percentage of fetal hemoglobin after birth is negatively correlated with the severity of ROP (105). The complete blood count, including low concentrations of hemoglobin, can be a simple screening indicator for ROP; this is especially true for the mean corpuscular hemoglobin (106). Mutations in a Wnt signaling receptor protein (FZD4) gene may be an indicator of ROP (107). High levels of neonatal hemoglobin A1C are a feasible biomarker for proliferative ROP, and low levels of A1C are a feasible biomarker for non-proliferative ROP (108). Elevated plasma E-selection levels and recombinant human erythropoietin (rhEPO) are independent risk predictors for the progression of ROP (109–111). The serine protease HTRA-1 is the basis of protection against preeclampsia-mediated ROP and prevents the occurrence of diseases (112). An increase in the concentration of lactate and a low value for the perfusion index may be early parameters that can be used to predict ROP (113). The lack of human chorionic gonadotropin (hCG) at 4 weeks after birth may be related to non-proliferative ROP (114). The increase in urinary N-terminal B-type natriuretic peptide (NT-proBNP) in the early stage of preterm infants (<30 weeks of gestational age) and the NT-proBNP/creatinine ratio can identify the risk of severe ROP (115, 116). However, the changes in NT-proBNP disappear in more mature infants (117). Cluster analysis showed that an early increase in the levels of Parkinson disease protein 7 (PARK7), vimentin, myeloperoxidase (MPO), CD69, and NF-κB essential modulator (NEMO) in plasma is related to a decrease in ROP risk. However, lower levels of tumor necrosis factor receptor superfamily member 4 (TNFRSF4) and higher levels of HER2 and galanin could predict the progression of ROP (118). In addition, a meta-analysis suggested that polymorphisms in the angiotensin-converting enzyme (ACE) I/D may be a genetic biomarker of an increased risk of ROP (119). The mean blur rate (MBR) was higher in OIR rats than in the control group, and it was significantly correlated with avascular area/total retinal area (%AVA) and retinal VEGF, therefore, MBR could be used to assess the severity of OIR (120).

## Comparison of Different Detection Methods

Although various biomarkers are associated with ROP, whether their different detection methods are easy and fast is an important part of determining the feasibility of their final application in premature infants. Premature infants are fragile, and thus, it is highly important to avoid invasive operations, such as obtaining aqueous humor. Less invasive examinations, such as blood tests, are reliable sources of biomarkers, and most of the potential biomarkers we summarized above are also obtained from blood. In addition to collecting plasma or serum, peripheral blood mononuclear cells (PBMCs) could be obtained during the blood examination. PBMCs are an essential part of the immune system. They are related to inflammatory cells and can release a large number of paracrine factors (121–124). They are potentially an important source of biomarkers that are related to cytokines and growth factors. In addition, saliva has been used to determine the level of melatonin in premature infants. No significant difference in the level of melatonin between serum and saliva has been demonstrated, and a high degree of correlation was observed (125). Perhaps these findings can provide new ideas for exploring biomarkers. It is convenient to collect urine. Urine is rich in metabolites and can reflect the total imbalance of all biochemical pathways in the body (126). It is worth further exploring the value of such non-invasive, pain-free and easy-to-obtain samples as potential monitoring, diagnostic and prognostic biomarkers in ROP.

## Conclusion

All over the world, the incidence of ROP is increasing in countries that have the technology to save premature infants (127, 128). If patients can be diagnosed in a timely manner, an effective treatment could restore vision. However, regrettably, the diagnosis of ROP depends on pediatric ophthalmologists with a great deal of clinical experience, and this diagnosis has a high degree of subjectivity and variability (129). It is easy to ignore abnormal conditions of the eyes because infants are unable to speak, but it could eventually lead to irreversible vision loss in some cases. Therefore, it is believed that the discovery of effective ROP biomarkers is very important. Reliable and easily available biomarkers will provide considerable information on diseases and aid in the development of new effective therapies (130). With the development of emerging laboratory medical technologies, microfluidic chips (131), proteomics (132) and single-cell technologies (133), also contribute to the exploration of biomarkers, which also deserve to be further revealed in ROP studies. In this review, we summarized several strong potential biomarkers including metabolites, cytokines and growth factors, ncRNAs, gut microbiota, iconography, oxidative stress biomarkers, etc. Since newborns are fragile, these markers should preferably be found in non-invasive and easily accessible samples, such as blood, urine and feces. Those studies and methods might contribute to the identification of effective biomarkers that shed light to the prediction and treatment of ROP.

## Author Contributions

YZ contributed to the conceptualization, design, and outline of this review. WT prepared the draft of the manuscript with tables and figures. BL, ZW, and JZ contributed to the literature search. YJ and SY contributed to the revision and editing. All authors have read and approved the final manuscript.

## Funding

This work was supported by Changsha Science and Technology Project (kq1907075) and the Fundamental Research Funds for the Central Universities of Central South University (No. 2021zzts1056).

## Conflict of Interest

The authors declare that the research was conducted in the absence of any commercial or financial relationships that could be construed as a potential conflict of interest.

## Publisher's Note

All claims expressed in this article are solely those of the authors and do not necessarily represent those of their affiliated organizations, or those of the publisher, the editors and the reviewers. Any product that may be evaluated in this article, or claim that may be made by its manufacturer, is not guaranteed or endorsed by the publisher.
